# Supplementary figures and images for: Vibrio cholerae O1 El Tor A1552 encodes two functional ornithine lipid synthases and induces ornithine lipid formation under low phosphate and under low salinity growth conditions
Source: PLoS One. 2025 Jun 24;20(6):e0316307. doi: 10.1371/journal.pone.0316307 (PMC12186959; doi:10.1371/journal.pone.0316307)

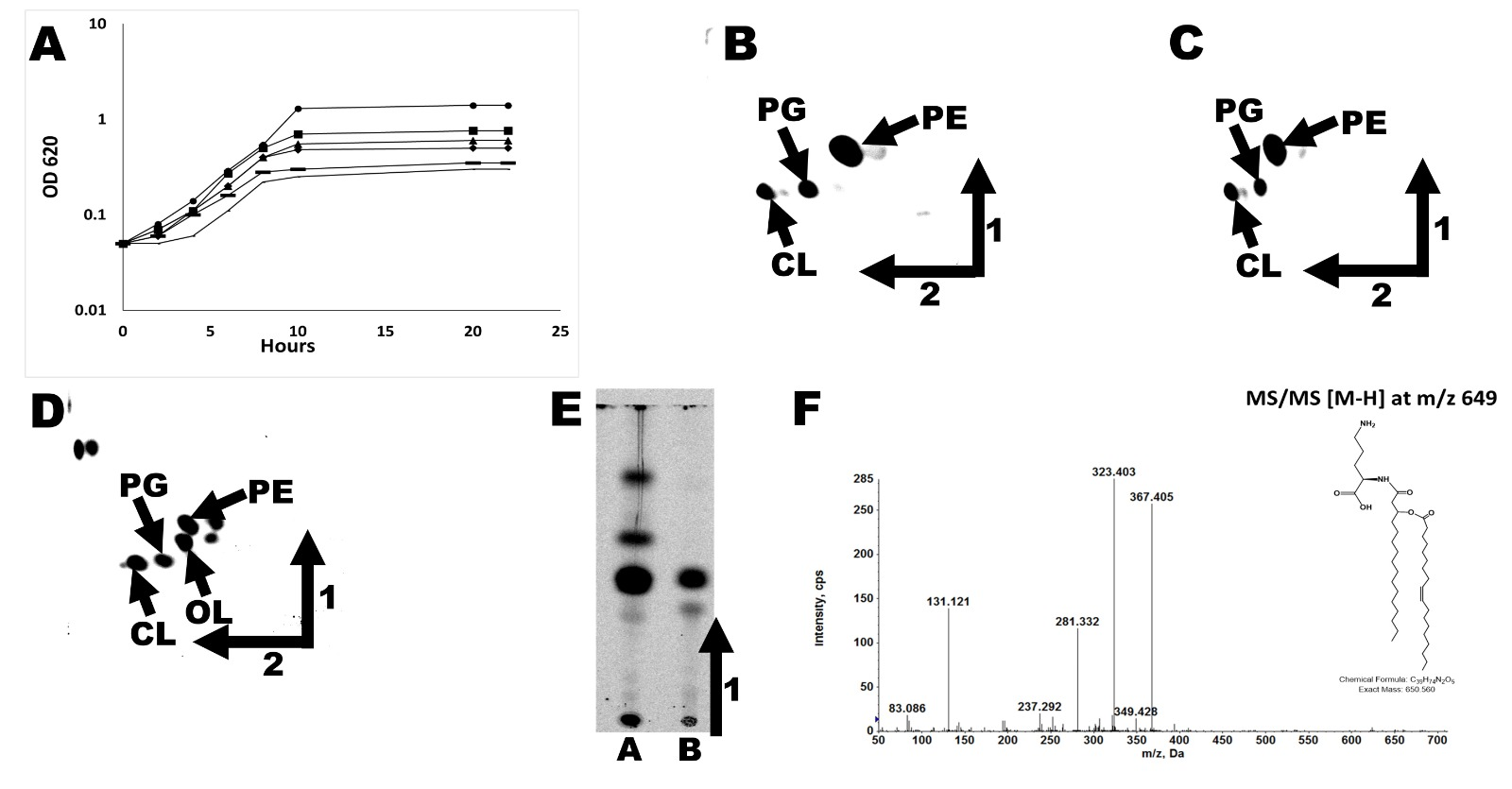

Supplement: S1 Fig — (A) Growth of wildtype V. cholerae in defined M9 medium with different phosphate concentrations. The symbols are defined in the inset. (B) Two-dimensional TLC separation of [14C]acetate-labeled lipids of wildtype V. cholerae grown in LB medium. (C) Two-dimensional TLC separation of [14C]acetate-labeled lipids of wildtype V. cholerae grown in M9 medium supplemented with 64 mM phosphate. (D) Two-dimensional TLC separation of [14C]acetate-labeled lipids of wildtype V. cholerae grown in M9 medium supplemented with 4.2 mM phosphate. (E) One-dimensional TLC separation of [14C]acetate-labeled (a) or [14C]ornithine-labeled (b) lipids of wildtype V. cholerae grown in M9 medium supplemented with 4.2 mM phosphate. The numbered arrows indicate the first and second dimensions of the chromatography separations. PG: phosphatidylglycerol; PE: phosphatidylethanolamine; CL: cardiolipin; OL: ornithine lipid. (TIF) [file pone.0316307.s001.tif]

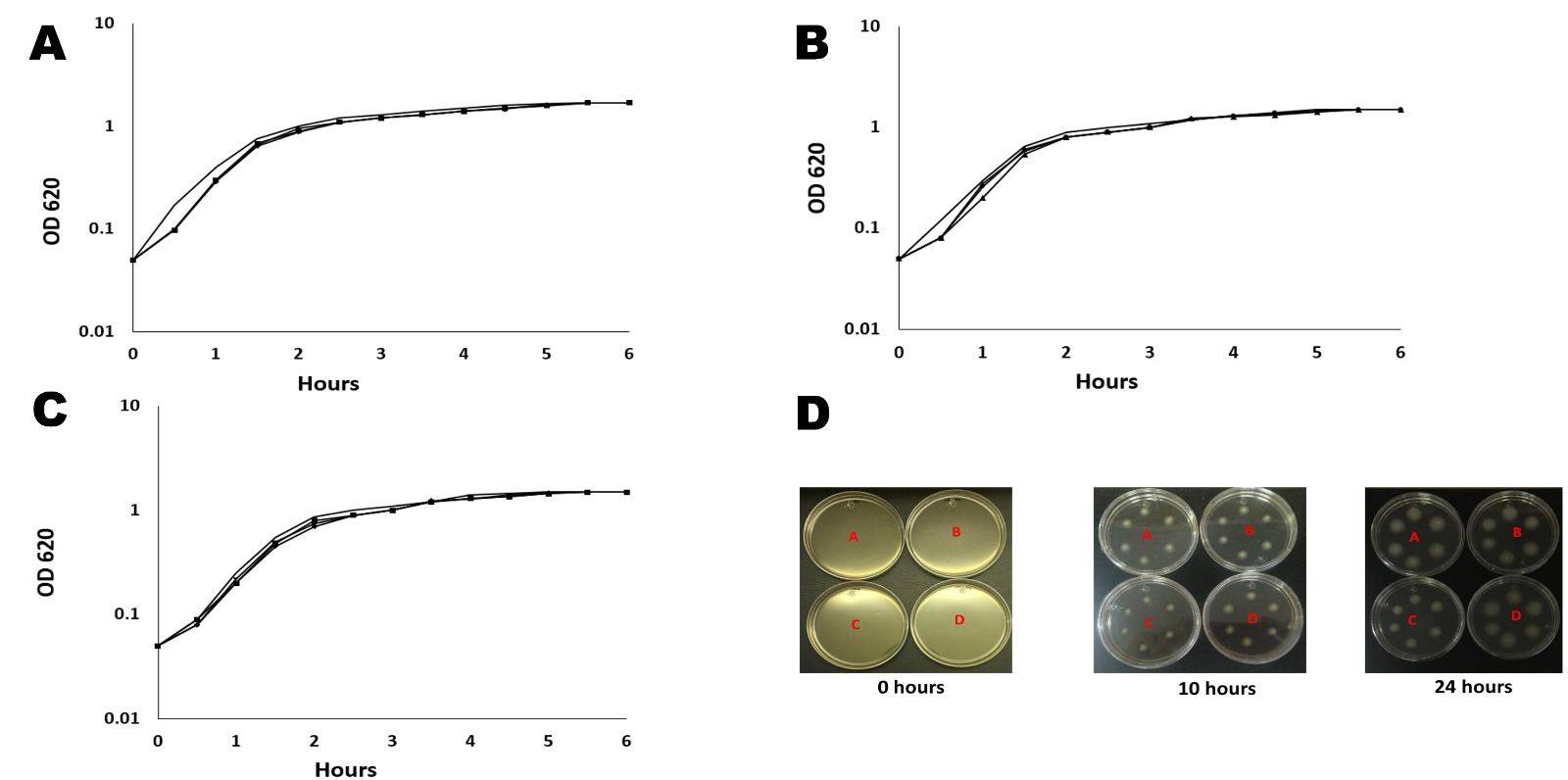

Supplement: S2 Fig — Single mutants ΔVC0489, ΔVCA0646, and double mutant 2M (ΔVC0489 and ΔVCA0646) were grown in LB medium with varying NaCl concentrations. Standard LB medium contains 10 g NaCl per liter, corresponding to approximately 0.17 M NaCl. (A) 0.15 M NaCl, (B) 0.075 M NaCl, (C) 0.0375 M NaCl. The symbols are defined in the inset of the figure. (D) Cells of the four strains were point-inoculated, and images were taken at 0 hours, 10 hours, and 24 hours. No differences could be observed between the four different strains. A: wildtype; B: ΔVC0489; C: ΔVCA0646; D: 2M (ΔVC0489 and ΔVCA0646). (TIF) [file pone.0316307.s002.tif]

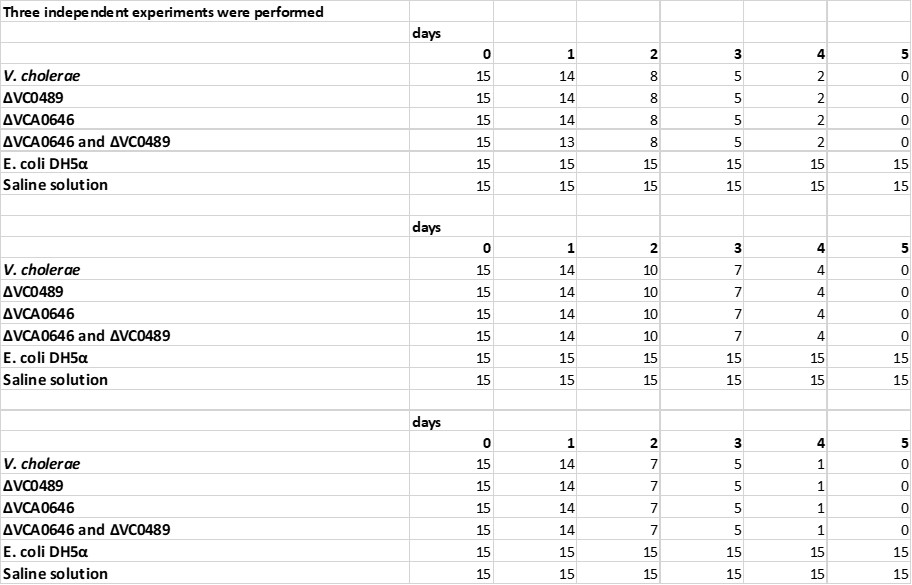

Supplement: S3 Fig — Assays were performed by the injection method with G. mellonella larvae, and 10 µl of control or bacterial suspensions were injected into the dorsal region of the third anterior abdominal segment of the larvae with a 31-gauge insulin syringe. Each bacterial suspension was analyzed using 15 insect larvae individually placed in Petri dishes without diet and incubated at 30 °C. Mortality was assessed every 24 hours for 5 days after injection. Three independent experiments were performed. (TIF) [file pone.0316307.s003.tif]
