# Supplementary material for: Vibrio cholerae O1 El Tor A1552 encodes two functional ornithine lipid synthases and induces ornithine lipid formation under low phosphate and under low salinity growth conditions
Source: PLoS One. 2025 Jun 24;20(6):e0316307. doi: 10.1371/journal.pone.0316307 (PMC12186959; doi:10.1371/journal.pone.0316307)

Supplementary Information for the manuscript “*Vibrio cholerae* O1 El Tor A1552 encodes two functional ornithine lipid synthases and induces ornithine lipid formation under low phosphate and under low salinity growth conditions.”
by Vences-Guzmán et al., 2025.

This document contains the original images of thin-layer chromatography experiments before cropping (if aplicable) and data used for the construction of the growth curves and the graph in Figure 5A.

The raw data for figures 4A, 4B, 5B and 5C are accessible in the supplementary tables 1 to 4.
Supplementary table 1: data for figure 4B
Supplementary table 2: data for figure 4C
Supplementary table 3: data for figure 5B
Supplementary table 4: data for figure 5C

Data for the construction of the growth curve presented in figure 2A. Growth curve presented in the manuscript is shown as a reference.

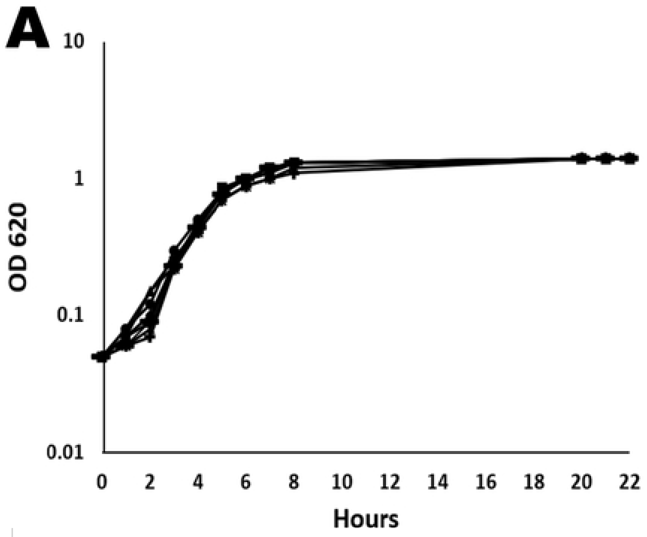

	0	1	2	3	4	5	6	7	8	20	21	22
WT	0.05	0.07	0.09	0.25	0.44	0.85	1	1.2	1.3	1.4	1.4	1.4
Δ646.pv	0.05	0.07	0.1	0.24	0.48	0.8	1	1.1	1.3	1.4	1.4	1.4
Δ489.pv	0.05	0.06	0.09	0.23	0.44	0.76	1	1.2	1.3	1.4	1.4	1.4
Δpho.pv	0.05	0.08	0.15	0.26	0.44	0.8	0.98	1.1	1.3	1.4	1.4	1.4
Δ646.p646	0.05	0.07	0.15	0.23	0.44	0.8	1	1.1	1.3	1.4	1.4	1.4
Δ489.p489	0.05	0.06	0.08	0.22	0.41	0.7	0.88	1	1.2	1.4	1.4	1.4
ΔPhoB.p489	0.05	0.08	0.12	0.3	0.5	0.82	1	1.2	1.3	1.4	1.4	1.4
Δ646, Δ489	0.05	0.06	0.07	0.3	0.41	0.7	0.88	1	1.1	1.4	1.4	1.4

	0	1	2	3	4	5	6	7	8	20	21	22
WT	0.05	0.1	0.12	0.36	0.6	0.9	1.1	1.3	1.4	1.5	1.5	1.5
Δ646.pv	0.05	0.1	0.11	0.3	0.52	0.88	1.1	1.2	1.4	1.5	1.5	1.5
Δ489.pv	0.05	0.1	0.15	0.23	0.5	0.76	1.1	1.2	1.4	1.5	1.5	1.5
Δpho.pv	0.05	0.1	0.15	0.26	0.5	0.8	0.98	1.1	1.4	1.5	1.5	1.5
Δ646.p646	0.05	0.1	0.15	0.36	0.47	0.86	1.1	1.3	1.4	1.5	1.5	1.5
Δ489.p489	0.05	0.1	0.12	0.36	0.5	0.7	0.88	1.1	1.3	1.5	1.5	1.5
ΔPhoB.p489	0.05	0.1	0.15	0.4	0.6	0.9	1.1	1.2	1.3	1.5	1.5	1.5
Δ646, Δ489	0.05	0.1	0.15	0.4	0.5	0.8	0.98	1.1	1.2	1.5	1.5	1.5

	0	1	2	3	4	5	6	7	8	20	21	22
WT	0.05	0.08	0.16	0.3	0.5	0.8	0.98	1.1	1.36	1.4	1.4	1.4
Δ646.pv	0.05	0.08	0.12	0.3	0.55	0.84	0.9	1.1	1.2	1.4	1.4	1.4
Δ489.pv	0.05	0.07	0.16	0.3	0.5	0.8	0.95	1.2	1.3	1.4	1.4	1.4
Δpho.pv	0.05	0.1	0.15	0.3	0.5	0.88	0.98	1.1	1.3	1.4	1.4	1.4
Δ646.p646	0.05	0.1	0.15	0.3	0.5	0.8	0.95	1.1	1.2	1.4	1.4	1.4
Δ489.p489	0.05	0.07	0.12	0.3	0.41	0.8	0.9	1	1.2	1.4	1.4	1.4
ΔPhoB.p489	0.05	0.08	0.16	0.36	0.55	0.87	0.98	1.2	1.3	1.4	1.4	1.4
Δ646, Δ489	0.05	0.1	0.15	0.36	0.4	0.9	1	1.1	1.2	1.4	1.4	1.4

Data for the construction of the growth curve presented in figure 2B. Growth curve presented in the manuscript is shown as a reference.

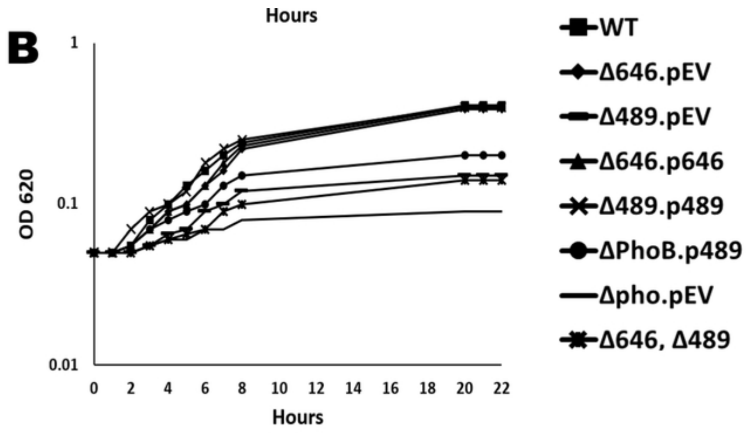

1		0	1	2	3	4	5	6	7	8	20	21	22
2	WT	0.05	0.05	0.055	0.08	0.1	0.13	0.16	0.2	0.24	0.41	0.41	0.41
3	Δ646.pEV	0.05	0.05	0.055	0.07	0.09	0.1	0.13	0.16	0.22	0.39	0.39	0.39
4	Δ489.pEV	0.05	0.05	0.05	0.055	0.065	0.07	0.09	0.1	0.12	0.15	0.15	0.15
5	Δpho.pEV	0.05	0.05	0.05	0.055	0.06	0.065	0.07	0.07	0.08	0.09	0.09	0.09
6	Δ646.p646	0.05	0.05	0.055	0.07	0.09	0.1	0.13	0.18	0.23	0.4	0.4	0.4
7	Δ489.p489	0.05	0.05	0.07	0.09	0.1	0.12	0.18	0.22	0.25	0.4	0.4	0.4
8	ΔPhoB.p489	0.05	0.05	0.055	0.07	0.08	0.09	0.1	0.13	0.15	0.2	0.2	0.2
9	Δ646, Δ489	0.05	0.05	0.05	0.055	0.06	0.065	0.07	0.09	0.1	0.14	0.14	0.14
10													
11		0	1	2	3	4	5	6	7	8	20	21	22
12	WT	0.05	0.05	0.055	0.089	0.11	0.14	0.17	0.2	0.22	0.4	0.4	0.4
13	Δ646.pEV	0.05	0.05	0.055	0.08	0.098	0.11	0.14	0.15	0.2	0.38	0.38	0.38
14	Δ489.pEV	0.05	0.05	0.053	0.06	0.07	0.08	0.09	0.1	0.11	0.16	0.16	0.16
15	Δpho.pEV	0.05	0.05	0.056	0.053	0.055	0.06	0.065	0.07	0.08	0.08	0.08	0.08
16	Δ646.p646	0.05	0.05	0.055	0.078	0.093	0.1	0.12	0.16	0.2	0.41	0.41	0.41
17	Δ489.p489	0.05	0.05	0.08	0.097	0.11	0.13	0.17	0.2	0.22	0.39	0.39	0.39
18	ΔPhoB.p489	0.05	0.05	0.055	0.075	0.085	0.094	0.11	0.13	0.14	0.19	0.19	0.19
19	Δ646, Δ489	0.05	0.05	0.05	0.055	0.066	0.07	0.08	0.09	0.1	0.15	0.15	0.15

	0	1	2	3	4	5	6	7	8	20	21	22
WT	0.05	0.05	0.07	0.09	0.12	0.15	0.16	0.22	0.24	0.42	0.42	0.42
Δ646.pEV	0.05	0.05	0.06	0.078	0.09	0.1	0.13	0.18	0.22	0.4	0.4	0.4
Δ489.pEV	0.05	0.05	0.05	0.058	0.065	0.077	0.09	0.11	0.12	0.14	0.14	0.14
Δpho.pEV	0.05	0.05	0.05	0.055	0.06	0.063	0.07	0.075	0.08	0.088	0.088	0.088
Δ646.p646	0.05	0.05	0.07	0.08	0.1	0.11	0.13	0.2	0.25	0.42	0.42	0.42
Δ489.p489	0.05	0.05	0.09	0.1	0.12	0.14	0.18	0.24	0.27	0.42	0.42	0.42
ΔPhoB.p489	0.05	0.05	0.06	0.072	0.08	0.087	0.1	0.13	0.15	0.2	0.2	0.2
Δ646, Δ489	0.05	0.05	0.05	0.055	0.06	0.065	0.072	0.09	0.11	0.146	0.146	0.146

The figure 2C is composed from the cropped images of two different thin-layer chromatogtaphy experiments.
The original experiment shown in the uncropped images was much larger and included strains and mutants not included in the present manuscript.
Red labeling in the original figures corresponds to the numbering in figure 2C.
Figure 2C from the manuscript is shown as a reference.

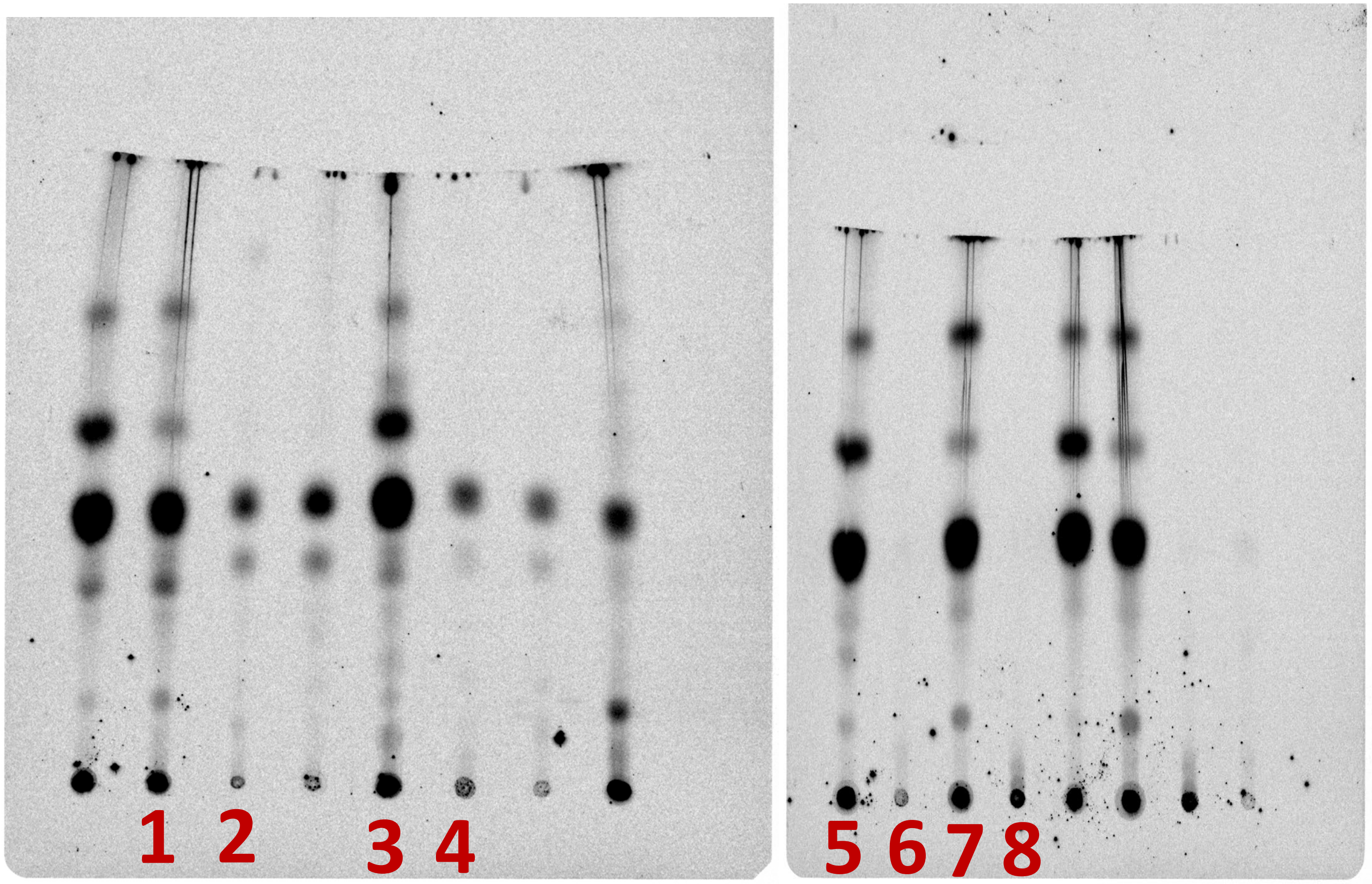

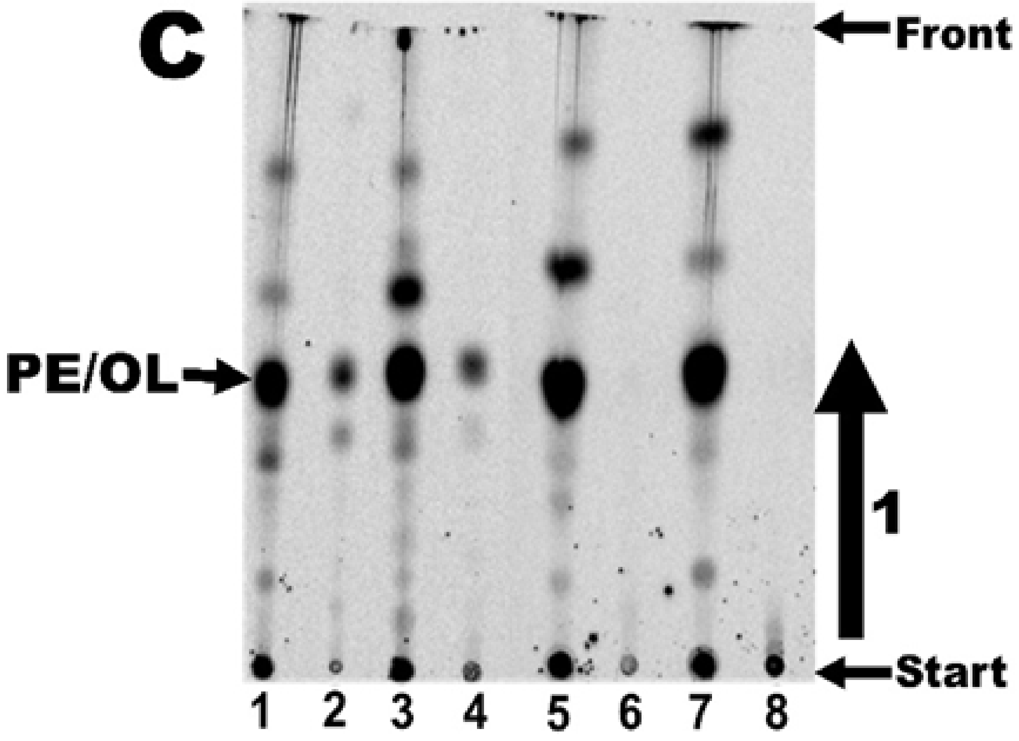

Uncropped images from thin-layer experiments used for the construction of figure 3 are shown.
Red numbers allow to make the reference between Original images and final figure.
Figure 3 from the manuscript is shown as a reference.
Here, the figures were cropped to keep figure 3 small.

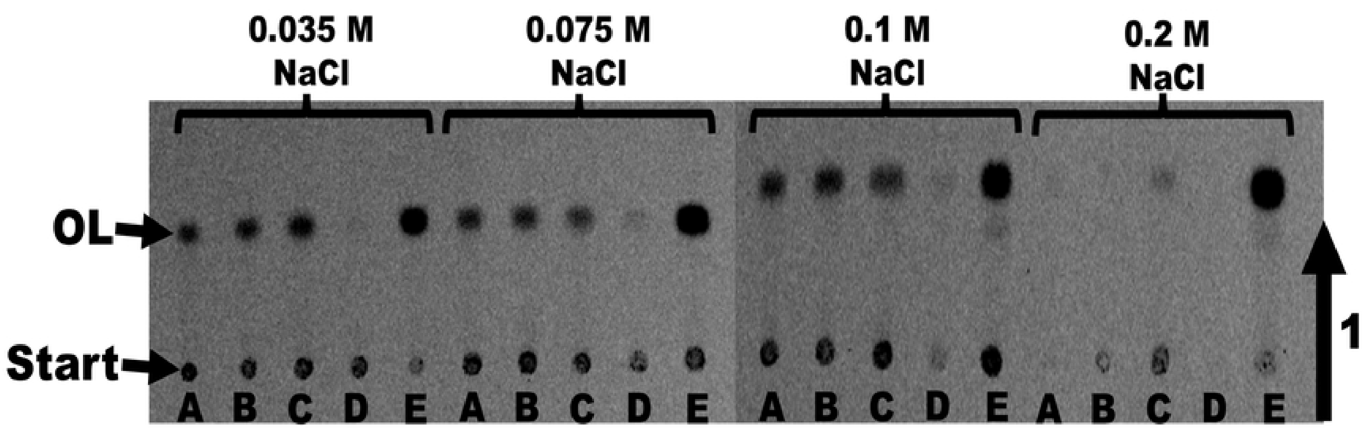

Figure 3

1

2

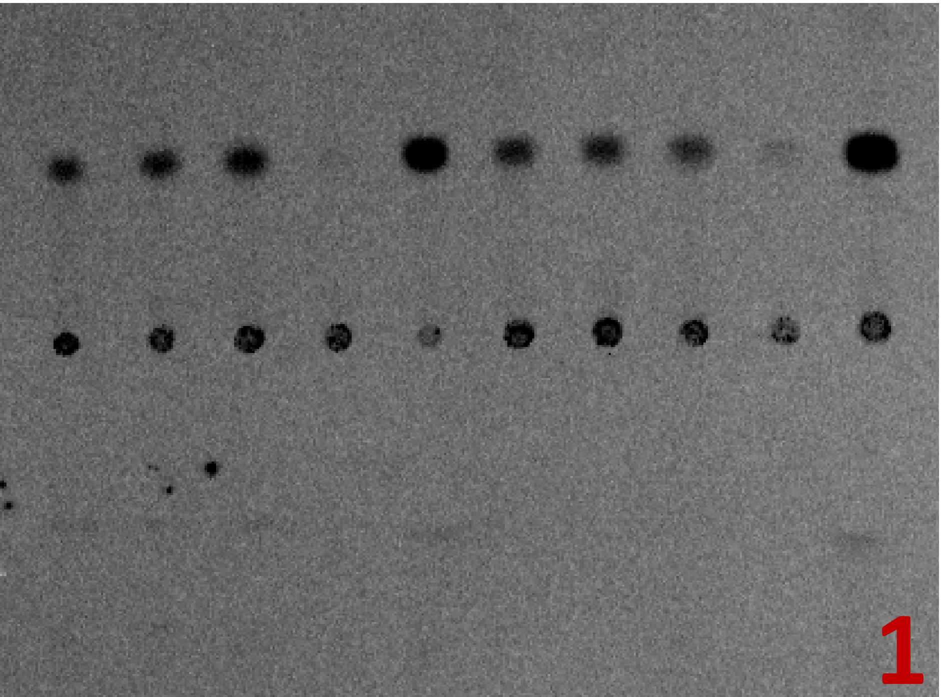

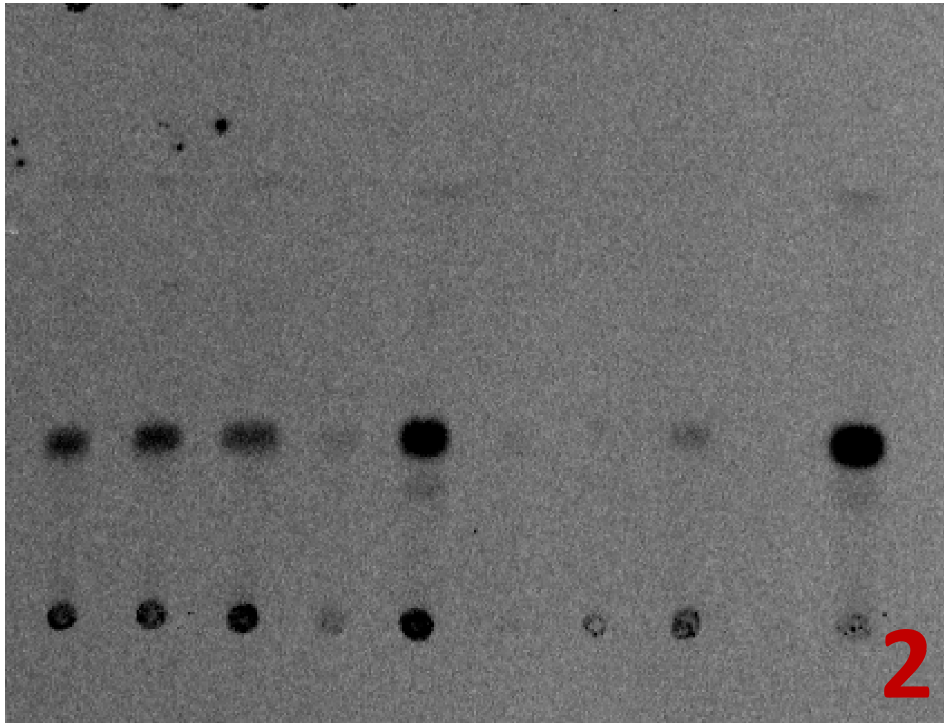

Data obtained from the experiment to compare the mutant strains with respect to polymyxin B resistance.
The experiment was performed in three replicates. Data used to construct figure 5A is shown.
Figure 5A from the manuscript is shown as a reference.

PB 300		
	0.2 M NaCl	0.037 M NaCl
WT	1.2	0.97
WT	1.1	0.9
WT	1.2	0.9
Δ489	1.12	0.8
Δ489	1.2	0.87
Δ489	1.2	0.81
Δ489.pVCO489	1.2	0.81
Δ489.pVCO489	1.2	0.85
Δ489.pVCO489	1.1	0.82
Δ646	1.2	1.61
Δ646	1.1	1.67
Δ646	1.2	1.6
Δ646.pVCA646	1.2	0.94
Δ646.pVCA646	1.2	0.93
Δ646.pVCA646	1.12	0.9

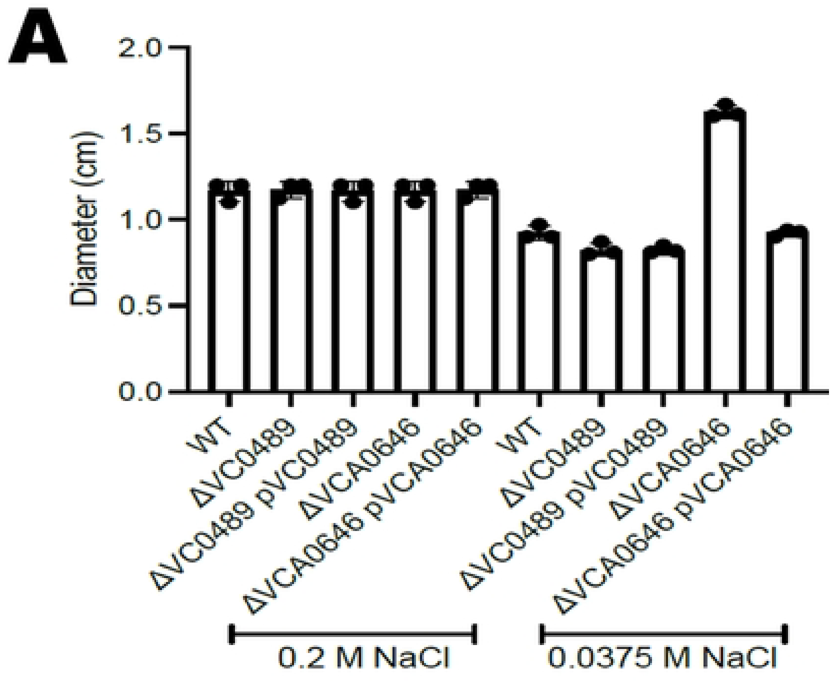

Data used for the construction of the growth curves presented in supplementary figure 1A.
The experiment was performed in three replicates.
Supplementary figure 1A is shown as a reference.

	0	2	4	6	8	10	20	22
WT 64 mM P	0.05	0.08	0.14	0.29	0.54	1.3	1.4	1.4
WT 12.8 mM P	0.05	0.07	0.11	0.27	0.5	0.7	0.76	0.76
WT 6.4 mM p	0.05	0.07	0.11	0.2	0.4	0.55	0.6	0.6
WT 4.2 mM P	0.05	0.06	0.11	0.2	0.4	0.48	0.5	0.5
WT 2.56 mM P	0.05	0.06	0.1	0.16	0.28	0.3	0.35	0.35
WT 1.28 mM P	0.05	0.05	0.06	0.11	0.22	0.25	0.3	0.3
	0	2	4	6	8	10	20	22
WT 64 mM P	0.05	0.09	0.16	0.31	0.53	1.25	1.41	1.41
WT 12.8 mM P	0.05	0.08	0.13	0.25	0.48	0.68	0.72	0.72
WT 6.4 mM p	0.05	0.07	0.12	0.2	0.41	0.55	0.65	0.65
WT 4.2 mM P	0.05	0.07	0.11	0.22	0.4	0.44	0.54	0.54
WT 2.56 mM P	0.05	0.06	0.11	0.18	0.28	0.35	0.4	0.4
WT 1.28 mM P	0.05	0.05	0.07	0.13	0.22	0.29	0.33	0.33
	0	2	4	6	8	10	20	22
WT 64 mM P	0.05	0.08	0.14	0.3	0.55	1.3	1.4	1.4
WT 12.8 mM P	0.05	0.07	0.11	0.29	0.5	0.7	0.74	0.74
WT 6.4 mM p	0.05	0.07	0.11	0.21	0.42	0.53	0.62	0.62
WT 4.2 mM P	0.05	0.06	0.11	0.2	0.39	0.46	0.51	0.51
WT 2.56 mM P	0.05	0.06	0.1	0.18	0.3	0.32	0.35	0.38
WT 1.28 mM P	0.05	0.05	0.06	0.12	0.25	0.28	0.3	0.3

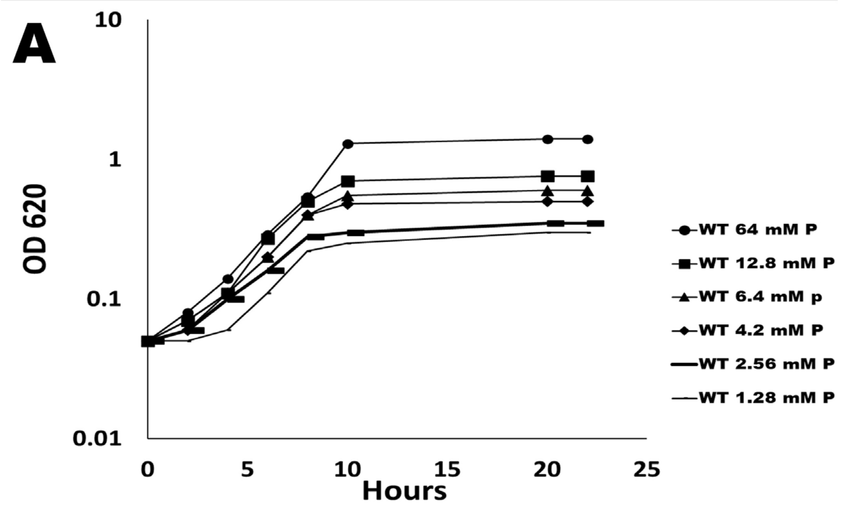

The original image of the thin-layer experiment was cropped in order to show only the lipid samples extracted from the wildtype strain and exclude the simples from other strains not included in the present manuscript. Same letters refer to the same lanes. Supplementary figure 1E is shown as a reference.

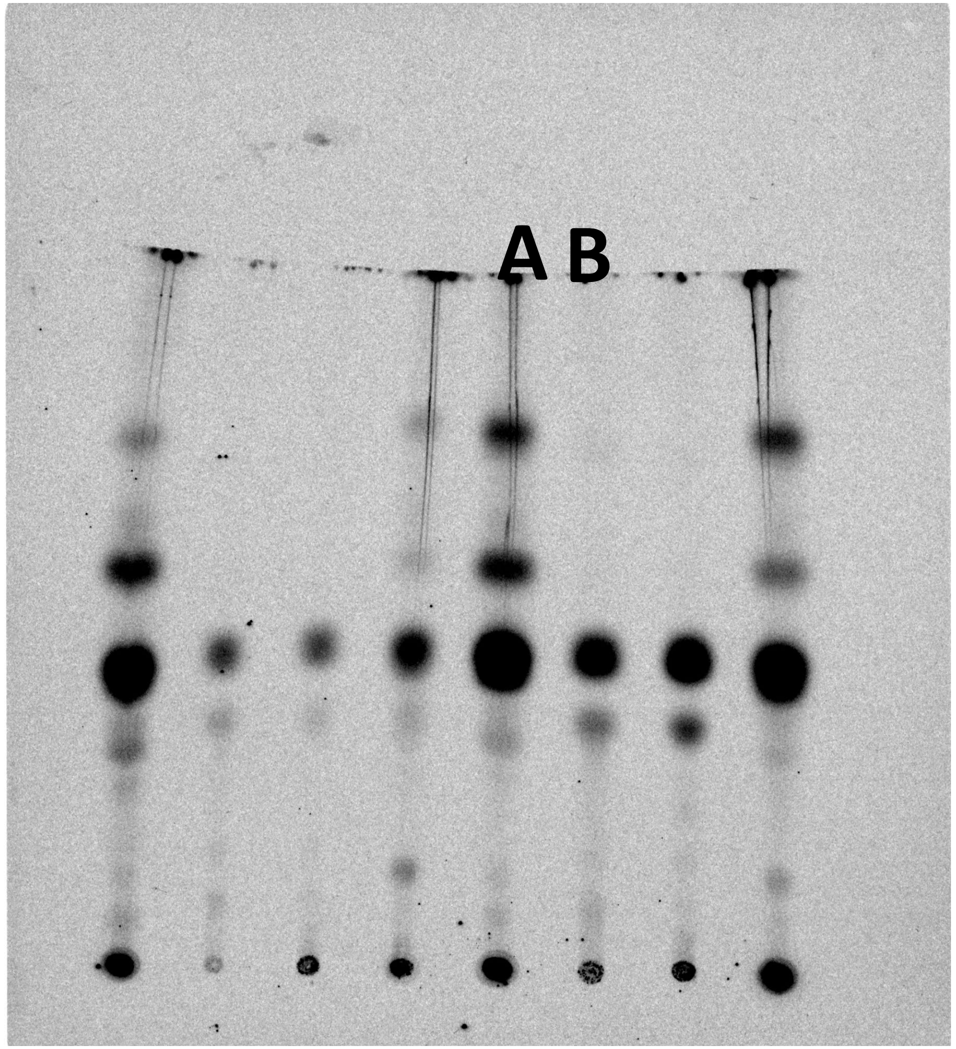

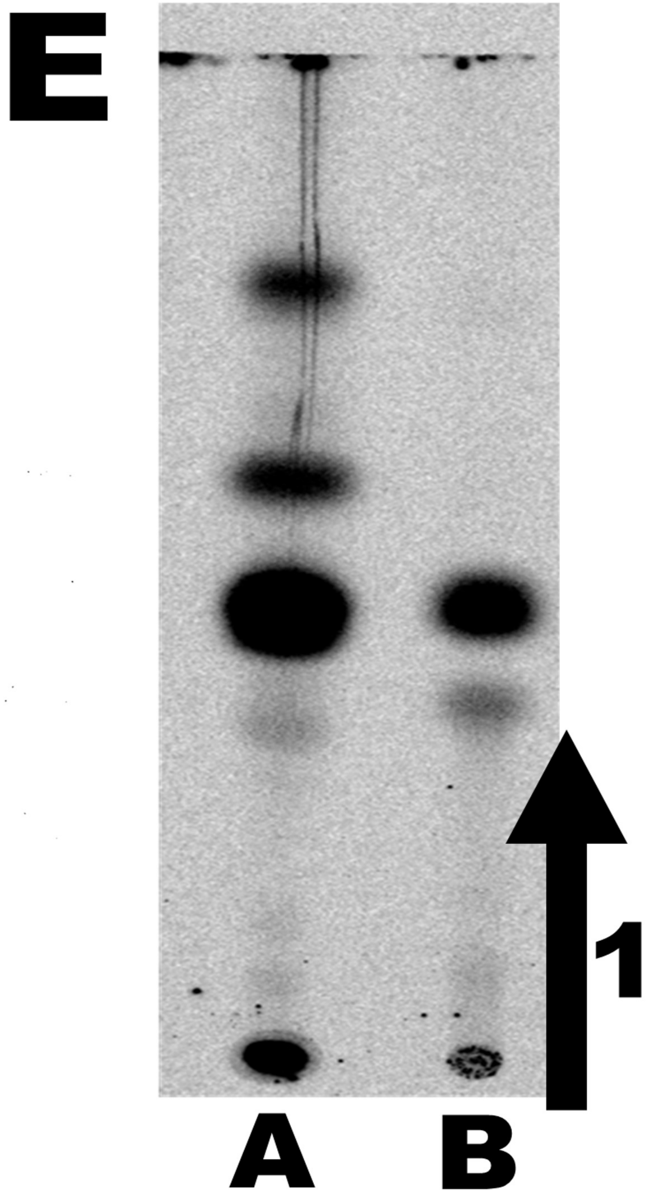

Data used for the construction of the growth curves shown in supplementary figure 2A. The experiment was performed in three replicates. As a reference, supplementary figure 2A is shown.

A	B	C	D	E	F	G	H	I	J	K	L	M	N
LB 0.15M NaCL													
	0	0.5	1	1.5	2	2.5	3	3.5	4	4.5	5	5.5	6
WT	0.05	0.17	0.4	0.75	1	1.2	1.3	1.4	1.5	1.6	1.65	1.7	1.7
ΔVC0489	0.05	0.099	0.3	0.66	0.96	1.1	1.2	1.3	1.4	1.5	1.6	1.7	1.7
ΔVCA0646	0.05	0.098	0.3	0.68	0.9	1.1	1.2	1.3	1.4	1.5	1.58	1.7	1.7
ΔVCA0646 ΔVC048	0.05	0.098	0.29	0.64	0.88	1.1	1.2	1.3	1.4	1.47	1.62	1.7	1.7
LB 0.15M NaCL													
	0	0.5	1	1.5	2	2.5	3	3.5	4	4.5	5	5.5	6
WT	0.05	0.19	0.42	0.78	1.1	1.26	1.33	1.42	1.52	1.61	1.68	1.72	1.72
ΔVC0489	0.05	0.11	0.32	0.69	1	1.11	1.22	1.33	1.42	1.52	1.65	1.71	1.71
ΔVCA0646	0.05	0.11	0.31	0.69	0.93	1.1	1.24	1.34	1.42	1.52	1.68	1.7	1.7
ΔVCA0646 ΔVC048	0.05	0.11	0.29	0.66	0.9	1	1.22	1.32	1.42	1.5	1.65	1.72	1.72
LB 0.15M NaCL													
	0	0.5	1	1.5	2	2.5	3	3.5	4	4.5	5	5.5	6
WT	0.05	0.18	0.41	0.77	1	1.22	1.31	1.4	1.55	1.63	1.65	1.72	1.72
ΔVC0489	0.05	0.1	0.31	0.68	1	1.12	1.23	1.32	1.45	1.51	1.64	1.7	1.7
ΔVCA0646	0.05	0.1	0.31	0.68	0.92	1.1	1.21	1.3	1.41	1.5	1.58	1.7	1.7
ΔVCA0646 ΔVC048	0.05	0.1	0.3	0.64	0.91	1.14	1.23	1.31	1.45	1.52	1.6	1.71	1.71

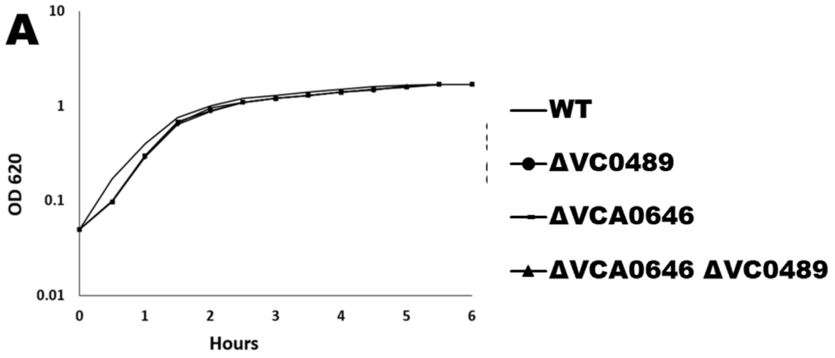

Data used for the construction of the growth curves shown in supplementary figure 2B. The experiment was performed in three replicates. As a reference, supplementary figure 2B is shown.

LB 0.075M NaCL													
	0	0.5	1	1.5	2	2.5	3	3.5	4	4.5	5	5.5	6
WT	0.05	0.12	0.3	0.65	0.9	1	1.1	1.2	1.3	1.4	1.5	1.5	1.5
ΔVC0489	0.05	0.08	0.26	0.6	0.8	0.9	1	1.2	1.3	1.38	1.44	1.5	1.5
ΔVCA0646	0.05	0.08	0.28	0.58	0.8	0.9	1	1.2	1.3	1.39	1.46	1.5	1.5
ΔVCA0646 ΔVC0489	0.05	0.08	0.2	0.54	0.8	0.9	1	1.23	1.28	1.32	1.42	1.5	1.5
LB 0.075M NaCL													
	0	0.5	1	1.5	2	2.5	3	3.5	4	4.5	5	5.5	6
WT	0.05	0.123	0.32	0.68	0.92	1.1	1.15	1.28	1.33	1.42	1.5	1.52	1.52
ΔVC0489	0.05	0.083	0.29	0.62	0.83	0.94	1.1	1.24	1.34	1.4	1.46	1.51	1.51
ΔVCA0646	0.05	0.08	0.29	0.57	0.81	0.92	1	1.22	1.33	1.4	1.46	1.5	1.5
ΔVCA0646 ΔVC0489	0.05	0.081	0.21	0.56	0.8	0.91	1.1	1.23	1.3	1.36	1.44	1.52	1.52
LB 0.075M NaCL													
	0	0.5	1	1.5	2	2.5	3	3.5	4	4.5	5	5.5	6
WT	0.05	0.121	0.3	0.66	0.9	1	1.12	1.24	1.31	1.4	1.5	1.5	1.5
ΔVC0489	0.05	0.09	0.3	0.61	0.82	0.92	1	1.21	1.3	1.4	1.5	1.5	1.5
ΔVCA0646	0.05	0.082	0.3	0.58	0.82	0.9	1.1	1.2	1.3	1.4	1.45	1.5	1.5
ΔVCA0646 ΔVC0489	0.05	0.08	0.2	0.55	0.81	0.94	1.1	1.23	1.32	1.4	1.42	1.5	1.5

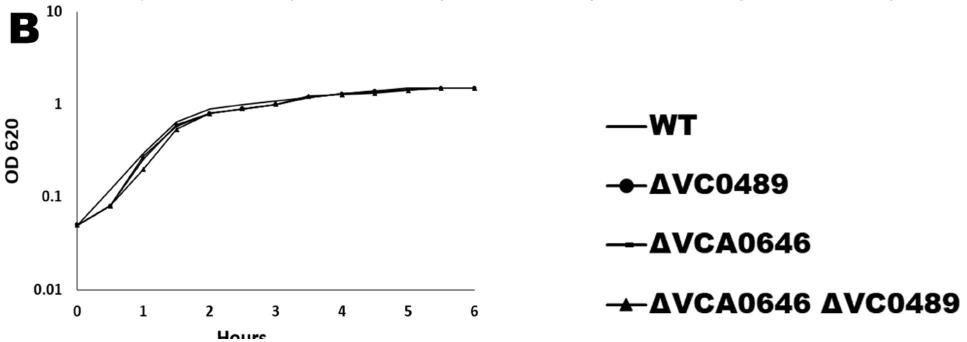

Data used for the construction of the growth curves shown in supplementary figure 2C. The experiment was performed in three replicates. As a reference, supplementary figure 2C is shown.

LB 0.037M NaCL													
	0	0.5	1	1.5	2	2.5	3	3.5	4	4.5	5	5.5	6
WT	0.05	0.09	0.25	0.55	0.86	1	1.1	1.2	1.3	1.35	1.45	1.5	1.5
ΔVC0489	0.05	0.09	0.2	0.48	0.8	0.9	1	1.2	1.3	1.35	1.45	1.5	1.5
ΔVCA0646	0.05	0.08	0.22	0.5	0.74	0.9	1	1.2	1.4	1.44	1.5	1.5	1.5
ΔVCA0646 ΔVC0489	0.05	0.08	0.2	0.45	0.7	0.9	1	1.23	1.3	1.4	1.47	1.5	1.5
LB 0.037M NaCL													
	0	0.5	1	1.5	2	2.5	3	3.5	4	4.5	5	5.5	6
WT	0.05	0.095	0.3	0.59	0.89	1	1.14	1.22	1.34	1.4	1.5	1.51	1.51
ΔVC0489	0.05	0.092	0.23	0.5	0.82	0.93	1.1	1.2	1.3	1.4	1.45	1.5	1.5
ΔVCA0646	0.05	0.081	0.25	0.52	0.78	0.96	1.1	1.23	1.41	1.48	1.51	1.51	1.51
ΔVCA0646 ΔVC0489	0.05	0.084	0.21	0.5	0.72	0.94	1	1.2	1.31	1.43	1.5	1.5	1.5
LB 0.037M NaCL													
	0	0.5	1	1.5	2	2.5	3	3.5	4	4.5	5	5.5	6
WT	0.05	0.093	0.26	0.54	0.88	0.96	1.1	1.2	1.3	1.34	1.5	1.5	1.5
ΔVC0489	0.05	0.091	0.22	0.47	0.81	0.92	1.1	1.2	1.31	1.35	1.4	1.5	1.5
ΔVCA0646	0.05	0.082	0.22	0.51	0.76	0.91	1.1	1.2	1.4	1.44	1.5	1.5	1.5
ΔVCA0646 ΔVC0489	0.05	0.081	0.22	0.48	0.71	0.91	1.1	1.2	1.32	1.4	1.5	1.5	1.5

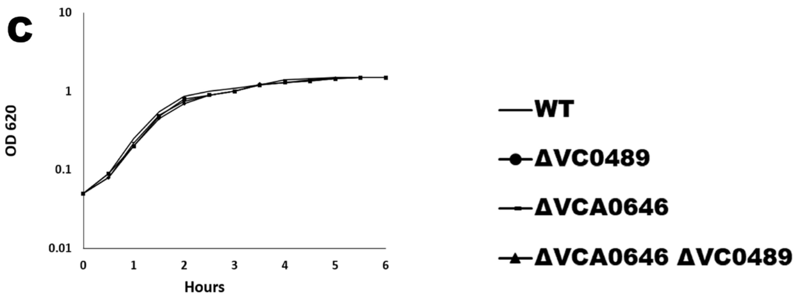

Supplement: S1 File — (PDF) [file pone.0316307.s004.pdf]
